# An Atypical Presentation of Disseminated Mucocutaneous Leishmaniasis Caused by *Leishmania major* In Iran

**Published:** 2018

**Authors:** Pedram NORMOHAMADPUR, Forugh GHAEDI

**Affiliations:** Razi Hospital, Vahdat Eslami Street, Tehran, Iran

**Keywords:** Leishmaniasis, Mucocutaneous, *Leishmania major*

## Abstract

Although leishmaniasis is an endemic disease in Iran the mucosal involvement is rare. Mucocutaneous leishmaniasis (MCL) mainly caused by *Leishmanial braziliensis* infection, reported with other *Leishmania* species such as *L. major*. Herein a 78 yr old man with MCL from Iran is presented who referred to Razi Hospital Dermatology Clinic, Tehran, Iran, for multiple ulcerative lesions on mid face skin, mucosa of upper lip and anterior fossa of nose, dorsal aspect of the hands and the posterior aspect of heels. Skin biopsy revealed necrotizing and granulomatous tissue pattern that suggested infection pathogenesis but the smear for leishmaniasis, *Mycobacterium* spp, and fungal elements was negative at first. In order to a positive PPD test, he was treated by anti-tuberculosis treatment. A month after starting drugs for tuberculosis, the prepared microscopical smears were positive for Leishman bodies this time. The skin biopsy revealed amastigote forms of *Leishmania* sp. and the PCR assay on specimens of lesions proved *L. major* as the principal pathogenic agent. There was good response to systemic treatment with meglumine antimoniate (Glucantime®) 3 gr per day until one week followed by 4.5 gr per day for another week. We forced to discontinue of drug because of cardiac toxicity at the end of 2^nd^ wk of treatment.

## Introduction

Leishmaniasis is a worldwide protozoal disease and is endemic in Iran. There are several clinical features but cutaneous and visceral leishmaniasis are the most common type of involvement in Iran as the same as the world (1,2).

An average annual incidence 32 cases per 100000 inhabitants for cutaneous leishmaniasis was reported in Iran based on the cross-sectional study from 1983 to 2012 (3).

The main etiological agents for cutaneous leishmaniasis are *L. major* and it is endemic in 17 out of 31 provinces of Iran, including Ilam that our patient is from there (4).

Cutaneous leishmaniasis caused by *L. major* is zoonotic and desert rodents (gerbils) are principal reservoir hosts of disease in various parts of Iran (5).

MCL is a destructive disease that affects the mucous membranes of the mouth, nose, pharynx, and larynx. Mucosal involvement is rare in Iran as well as its low prevalence in the world (6).

This type of leishmaniasis mainly caused by *L. braziliensis* infection, although it has occasionally been reported with other *Leishmania* species like *L.* *major* (7–9), *L.* *tropica* (7, 10), *L. Infantum* (11,12).

## Case report

**Fig. 1:**
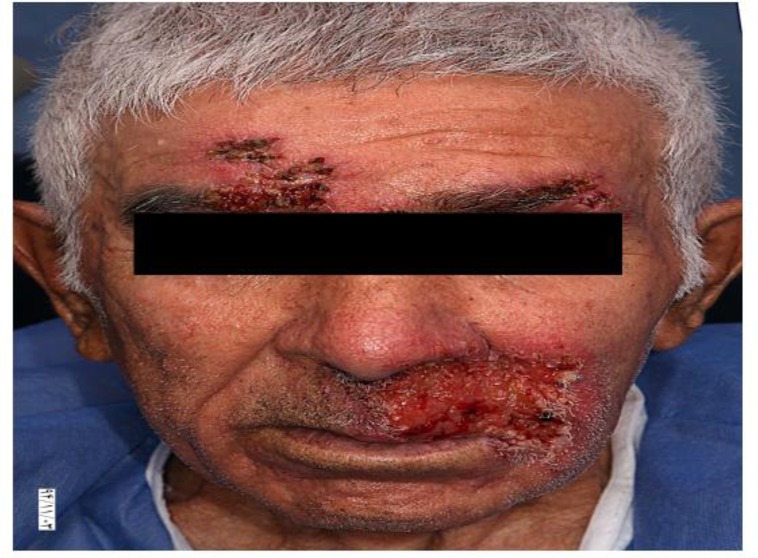
Mucocutaneous lesions on the face

**Fig. 2:**
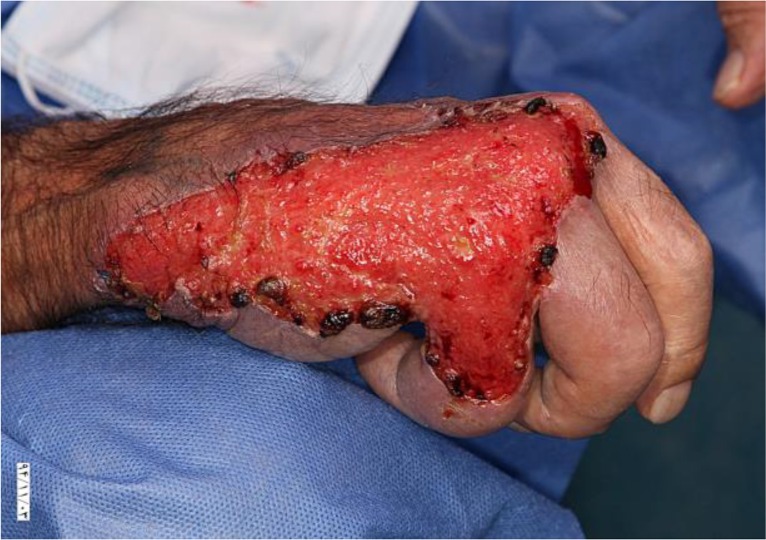
Ulcerative lesion on the right hand

A 78 yr old man from a rural area at the western of Iran referred to Razi Hospital Dermatology Clinic, Tehran for multiple ulcerative and exudative lesions on mid face, dorsal aspect of hands and the posterior aspect of heels (Fig. 1, 2).

Informed consent was taken from the patient.

Lesions initiated three years before with papules on the dorsal aspect of the hands then progressively enlarged above the upper lip, anterior portion of the nasal fossa, above the eyebrows and heels and became ulcerative.

During the past 3 yr, the lesions of hands were so developed that destroyed the tendons and soft tissue of fifth finger in the right hand so led to amputation of this finger. There was no history of comorbid condition, drug consumption, systemic symptoms, weight loss, fever, lymphadenopathy, hepatosplenomegaly or any signs of systemic involvement in physical examination and laboratory survey.

Multiple treatments in order to some heterogenic diagnosis such as pyoderma gangrenosum, sarcoidosis, and leishmaniasis were tried without any improvement in lesions.

In Razi Hospital Dermatology Clinic, initial Skin biopsy revealed necrotizing and palisading granulomatous tissue pattern that suggested infections etiology but the smear of lesions for fungal and mycobacteria and *Leishmania* was negative. In order to result of PPD test with 27 mm induration, anti-tuberculosis treatment including isoniazid, rifampin, ethambutol, and pyrazinamide was started.

A month after initiating drugs for tuberculosis, the smear of leishmaniasis repeated that was positive this time, the second biopsy revealed pseudoepitheliomatous hyperplasia and infiltration of the dermis by mixed inflammatory cells and Leishman bodies compatible with leishmaniasis.

**Fig. 3:**
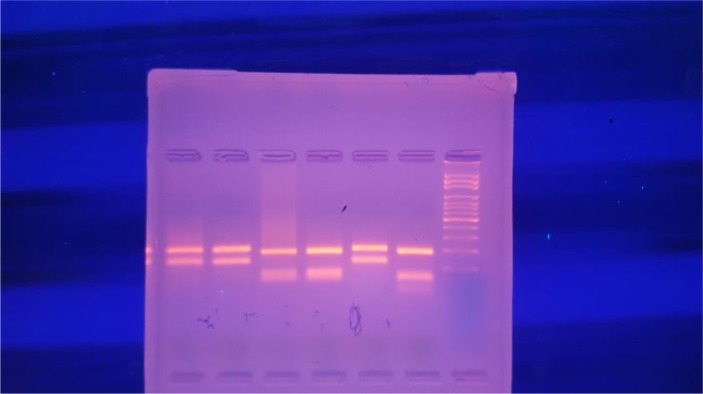
The PCR–RFLP on specimens of lesions

Restriction fragment length polymorphism (RFLP) PCR was carried out on DNA extraction was carried out with QIAGEN Kit according to the manufacturer’s instruction using two primers, LITSR (5-GTG CAG GAT CAT TTT CCG ATG) and L5.8s: 5-TGA TAC CAC TTA TCG CAC TT was designed for LTS1–PCR and cutting with and HaeIII enzyme. Positive controls containing DNA of *L. major*, *L. tropica* and a negative control containing distilled water were included. The PCR–RFLP on specimens of lesions proved *L. major* as the pathogenic agent (13, 14)(Fig. 3).

From the left side, the 2 bands show *L. major*, the second 2 bands show *L. tropica*, the 5^th^ one is the specimens of lesions that compatibles with *L. major* and the last one show *L. tropica*.

**Fig.4:**
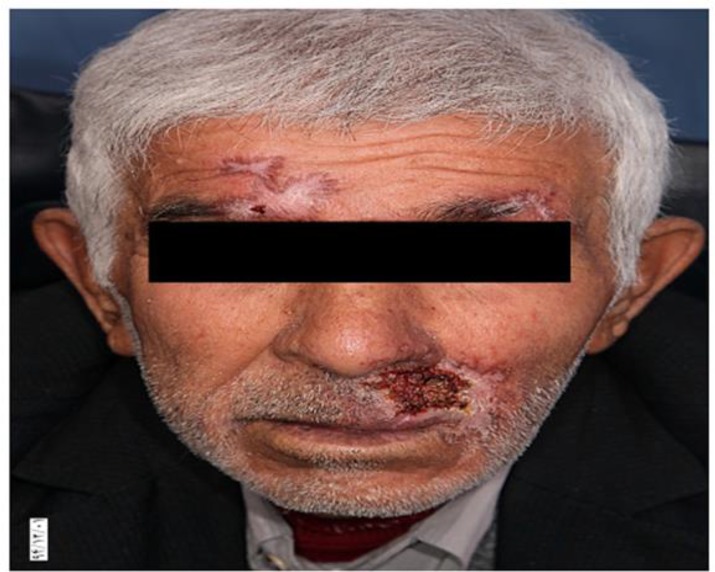
Significant treatment of lesion by meglumine antimoniate

**Fig. 5:**
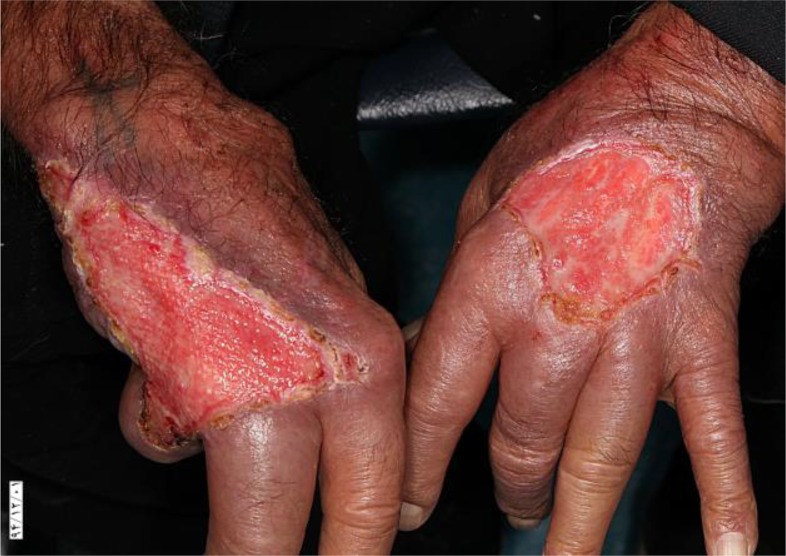
Significant treatment of lesion by meglumine antimoniate

Treatment initiated with 3 gr per day of meglumine antimoniate (Glucantime®) until 1 wk followed by 4.5 gr per day for another week, in spite of good response to meglumine antimoniate after 2 wk of treatment we forced to discontinue of drug because of cardiac toxicity (Figs. 4, 5).

The patient was informed about publishing his figures whit covering eyes.

## Discussion

Although leishmaniasis is an endemic disease in Iran, the mucosal involvement is a rare condition. Furthermore, there are a few reports of mucosal leishmaniasis with *L. major* in Iran (7–9, 15).

Shirian et al evaluated clinical features of 11 Iranian patients with mucosal leishmaniasis (15). They demonstrated pathogenesis of mucosal involvement in leishmaniasis was different in order to the causative species. *L. infantum* and *L. tropica* induced primary mucosal lesions without former skin involvement but *L. major* was the causative agent of secondary (metastatic) mucosal leishmaniasis. Lymphatic or hematogenous dissemination of parasites via the affected skin may cause localization of the disease in the nasal, oral, and pharyngeal mucosa. Compatible with Shirian et al observations, in our case, the primary lesions were cutaneous and the mucosal lesions were developed from distribution of skin lesions.

Furthermore Shirian et al observed *L. major* was the most causative agent in the lesions of nose, gingival, hard and soft palates and *L. tropica* was detected from the gingival and lower lip lesions. *L. infantum* was isolated from two cases with sever involvement of epiglottis and laryngeal mucosa (15). In our case, the mucosa membrane of the nose and upper lip was involved, compatible with the pathogenic manner of *L. major* that Shirian et al observed (15).

In the background of an immunosuppression such as HIV infection, renal transplantation, and drug-induced immunosuppression, disseminated cutaneous lesion with *L. major* is probably (16,17). However, in our patient there was no evidence for immunosuppression. Four cases of disseminated cutaneous leishmaniasis with *L. major* were reported (18). As none of them had no immunosuppressive state that explained the extent of lesions, they indicated the genomic characteristics of *Leishmania* parasites may be a significant role in *Leishmania* pathogenicity. Because of increasing trips and migration between endemic and non-endemic areas, in recent years, change in distribution of the parasite is occurred (19) and it is possible to produce new hybrid of *Leishmania* with different pathogenicity (20, 21).

DNA based molecular methods suggest for true identification of the causative agent of leishmaniasis for appropriate treatment, control, and prevention. Our patient was from Ilam Province located in the west of Iran near the Iraq border and their theory about changing in the pathogenicity of *L. major* is possible.

Apart of the rare manifestation of this patient as a disseminated cutaneous and mucosal form of leishmaniasis with *L. major*, an interesting point of this case is possible co-infection with *Mycobacterium tuberculosis* in order to a strong positive PPD test and granulomatous pattern of skin biopsy. Although we could not succeed to detect acid-fast basil from the lesion but the parasite of *Leishmania* was detected after treatment of tuberculosis, therefore, secondary infections can interfere with diagnosis of leishmaniasis so in infected cases, it is necessary to return evaluate leishmaniasis after a period of appropriate antibiotic therapy if a strong suspicion to leishmaniasis is propounded.
